# Rapid Detection of Carbapenem Resistance in *Acinetobacter baumannii* Using Matrix-Assisted Laser Desorption Ionization-Time of Flight Mass Spectrometry

**DOI:** 10.1371/journal.pone.0031676

**Published:** 2012-02-16

**Authors:** Marie Kempf, Sofiane Bakour, Christophe Flaudrops, Meryem Berrazeg, Jean-Michel Brunel, Mourad Drissi, Esma Mesli, Abdelaziz Touati, Jean-Marc Rolain

**Affiliations:** 1 Aix-Marseille-Université, Unité de Recherche sur les Maladies Infectieuses et Tropicales Émergentes (URMITE), CNRS-IRD-INSERM UMR 6236, Méditerranée Infection, Faculté de Médecine et de Pharmacie, Marseille, France; 2 Département de Microbiologie, FSNV, Université A/MIRA de Béjaia, Béjaia, Algeria; 3 Laboratoire Antibiotiques, Antifongiques, Physico-chimie, Synthèse et Activité Biologique (LAPSAB), Université Abou Bekr Belkaïd-Tlemcen, Tlemcen, Algeria; Charité-University Medicine Berlin, Germany

## Abstract

Rapid detection of carbapenem-resistant *Acinetobacter baumannii* strains is critical and will benefit patient care by optimizing antibiotic therapies and preventing outbreaks. Herein we describe the development and successful application of a mass spectrometry profile generated by matrix-assisted laser desorption ionization-time of flight (MALDI-TOF) that utilized the imipenem antibiotic for the detection of carbapenem resistance in a large series of *A. baumannii* clinical isolates from France and Algeria. A total of 106 *A. baumannii* strains including 63 well-characterized carbapenemase-producing and 43 non-carbapenemase-producing strains, as well as 43 control strains (7 carbapenem-resistant and 36 carbapenem-sensitive strains) were studied. After an incubation of bacteria with imipenem for up to 4 h, the mixture was centrifuged and the supernatant analyzed by MALDI-TOF MS. The presence and absence of peaks representing imipenem and its natural metabolite was analyzed. The result was interpreted as positive for carbapenemase production if the specific peak for imipenem at 300.0 m/z disappeared during the incubation time and if the peak of the natural metabolite at 254.0 m/z increased as measured by the area under the curves leading to a ratio between the peak for imipenem and its metabolite being <0.5. This assay, which was applied to the large series of *A. baumannii* clinical isolates, showed a sensitivity of 100.0% and a specificity of 100.0%. Our study is the first to demonstrate that this quick and simple assay can be used as a routine tool as a point-of-care method for the identification of *A. baumannii* carbapenemase-producers in an effort to prevent outbreaks and the spread of uncontrollable superbugs.

## Introduction

Carbapenems, the most common of which are imipenem and meropenem, are among the drugs of choice for the treatment of nosocomial infections due to *Acinetobacter baumannii* [1]. However, their efficacies are increasingly becoming compromised because of the worldwide emergence of resistant isolates [2]–[4]. This resistance is principally caused by the production of carbapenemases, enzymes that are grouped into the following four classes according to their molecular structure: 1) Ambler class A β-lactamases, which are partially inhibited by clavulanic acid; 2) Ambler class B β-lactamases, which are also referred to as metallo-β-lactamases (MBL) because of the presence of a Zn^2+^ ion within the active site; 3) Ambler class C β-lactamases; and 4) Ambler class D β-lactamases, which are also referred to as oxacillinases (or OXA-type β-lactamases) and are serine-site enzymes [5], [6]. For *A. baumannii*, carbapenem resistance is principally mediated by the production of oxacillinases, mainly the *bla*_OXA-23-like_, *bla*_OXA-24-like_ and *bla*_OXA-58-like_ gene products [7]–[13]. Each of these enzymes is able to hydrolyze the amide bond of the β-lactam ring of carbapenems [6].

Currently, there is no standardized direct phenotypic method for the detection of *A. baumannii* carbapenemases in routine microbiological laboratories, although there are indirect methods that are based on the ability of some compounds to inhibit carbapenemases. For example, MBLs are susceptible *in vitro* to inhibition by EDTA, but phenotypic MBL detection using the E-test containing imipenem with or without EDTA is not reliable because there can be false positives [14]. OXA-type carbapenemases are usually susceptible to NaCl inhibition, but some do not hydrolyze oxacillin or cloxacillin [5]. In addition, NaCl-mediated *in vitro* inhibition of their activity is not always observed, and moreover, OXA-positive clinical isolates often express additional non-OXA-type carbapenemases. PCR-based methods remain the optimal tool for the identification of OXA-type carbapenemases, but the main disadvantages of such technologies include cost, the requirement for trained personal, and the inability to detect novel carbapenemase genes [15].

Thus, there is an urgent need for a rapid, sensitive, specific and inexpensive test for the detection of carbapenemase activity. The rapid detection of resistant strains is critical and will benefit patient care by hastening diagnoses, optimizing therapy with antibiotics and preventing outbreaks. Recently, it was demonstrated that the detection of carbapenemase activity in *Enterobacteriaceae* and *Pseudomonas aeruginosa* could be achieved through the detection of the ertapenem and meropenem molecules and their natural degradation products using matrix-assisted laser desorption ionization-time of flight (MALDI-TOF) [16], [17]. Herein, we describe the development and successful application of a mass spectrometry profile generated by matrix-assisted laser desorption ionization-time of flight (MALDI-TOF) that utilized the imipenem antibiotic for the detection of carbapenem resistance in a large series of *A. baumannii* clinical isolates from France and Algeria.

## Results

### Identification of bacteria, antibiotic susceptibility testing and molecular characterization of carbapenemase encoding genes

All 106 isolates were identified as *A. baumannii* using MALDI-TOF MS, with score values above 2.2 for all strains. Results of antibiotic susceptibility testing showed that among the 106 *A. baumannii* strains, 63 were found to be resistant to imipenem (MICs >8 mg/L confirmed using Etest). All 63 strains were checked for the presence of carbapenemase encoding genes and results showed that 57 of them harbored a *bla*_OXA-23_ gene (17 were isolated in Marseille and 40 in Algeria), 3 harbored a *bla*_OXA-24_ gene (isolated in Algeria) and 3 a *bla*_OXA-23+_ *bla*_OXA-24_ gene (isolated in Algeria) (Table 1).

**Table 1:** Characterization of the 149 bacterial strains analyzed and data summary of imipenem hydrolysis assay utilizing MALDI-TOF MS.

	MALDI-TOF analysis			
Strain type: No. of isolates				
(Location of isolates [No.of isolates])	Range of the ratio of the area of imipenem/metabolite based on the time of incubation		No. of isolates detected as carbapenemase producers (disappearance of the peak at 300 m/z or ratio between the peak for imipenem and its metabolite being <0.5) based on the time of incubation	
	2 h	4 h	2 h	4 h
**Carbapenem-resistant strains (70)** (imipenem MIC >8 mg/L)			67	70
*K. pneumoniae* KPC: 1	<0.01	<0.01	1	1
*K. pneumoniae* NDM-1: 2	<0.01	<0.01	2	2
*P. aeruginosa* VIM: 2	<0.01	<0.01	2	2
*P. aeruginosa* IMP: 2	<0.01	<0.01	2	2
*A.baumannii* bla_OXA23-like_: 57 (Marseille [17], Algeria [40])	<0.01–1.77	<0.01–0.23	54	57
*A.baumannii* bla_OXA24-like_: 3 (Algeria)	<0.01	0.02–0.04	3	3
*A.baumannii* bla_OXA23-like_+bla_OXA24-like_: 3 (Algeria)	<0.01–0.48	<0.01–0.06	3	3
**Carbapenem-susceptible strains (79)** (imipenem MIC £2 mg/L)			0	0
*K. pneumoniae* ESBL: 31 (Algeria)	ND	0.64–14.84	0	0
*K. pneumoniae* non ESBL: 4 (Algeria)	ND	1.24–5.82	0	0
*Escherichia coli* ATCC 25922 (1)	1.64	1.17	0	0
*A. baumannii*: 43 (Marseille [1], Algeria [42])	0.61–12.86	0.97–4.96	0	0

### Standardization and internal calibration of the Ultraflex I mass spectrometer with imipenem solution

**Figure 1:**
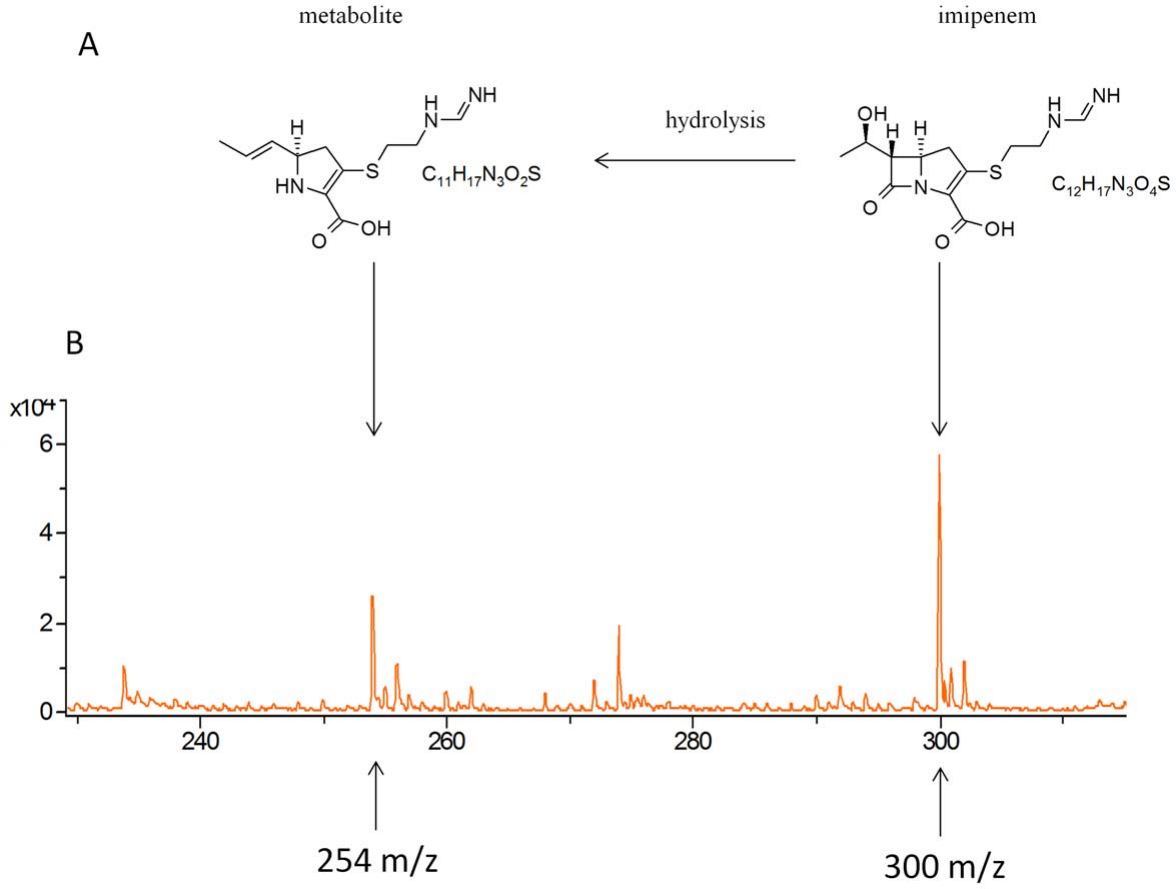
MALDI-TOF MS analysis of imipenem. (A) Imipenem and its natural degradation product. (B) Mass spectra of imipenem and its natural degradation product as determined using the Ultraflex mass spectrometer.

Theoretical atomic masses of imipenem (C_12_H_17_N_3_O_4_S) and its natural metabolite (C_11_H_17_N_3_O_2_S) (Figure 1A) were calculated using ISIS Draw software and were at 299.35 g/mol and 255.35 g/mol, respectively. The three matrix described in methods were tested with 0.45% NaCl and then combined with imipenem. The matrix containing acetone with ethanol and TFA provided spectra with the most useful data, i.e., without additional background peaks at the imipenem and imipenem natural metabolite peaks positions. Therefore, this matrix was selected for all further tests. Firstly we established and standardize the mass spectrum of pure imipenem in order to calibrate the mass spectrometer to check for the presence and reproducibility of detection of both imipenem and its natural metabolite. The characteristic mass spectrum of pure imipenem consists of both a main peak at 300.0+/−0.2 m/z for imipenem (n = 200 experiments) and a weak peak at 254.0+/−0.1 m/z for the natural metabolite (n = 200 experiments) (Figure 1B). In order to standardize our assay we decide to include for each bacterial isolate tested a ratio calculation between area under curve of imipenem and its metabolite allowing a precise and reproducible internal control of the experiments. Finally, we have checked for autodegradation of imipenem to ensure that the compound was stable during the experiments and we show that the presence of imipenem was stable during 6 hours of incubation with a ratio between area under curve of imipenem and its metabolite being always >1 (Figure S1).

### Imipenem hydrolysis assay

**Figure 2:**
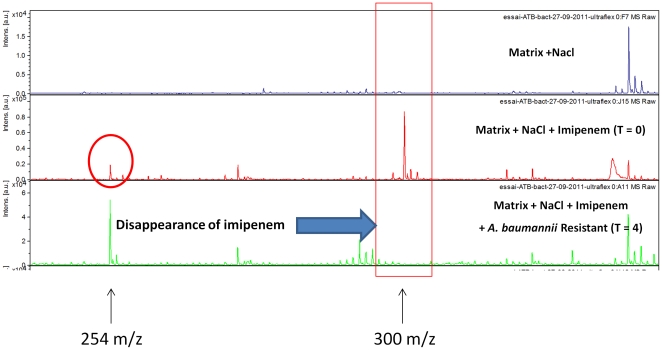
Mass spectra of the imipenem hydrolysis assay with a carbapenem-resistant *A. baumannii* strain. Incubation at 37°C during 4 h; NaCl 0.45%; imipenem concentration 0.25 mg/mL. Units of the *x* axis represent the mass per charge in Daltons [m/z (Da)] and that of the *y* axes, the relative intensity (a.u., arbitrary units).

**Figure 3:**
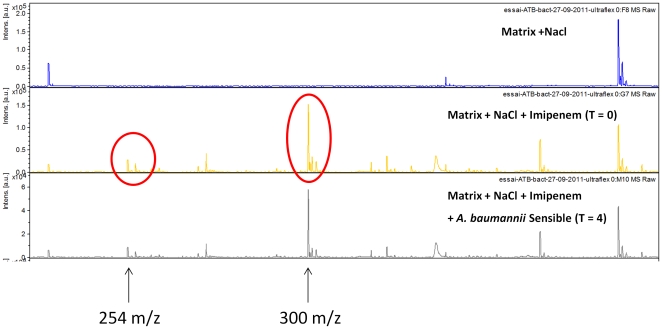
Mass spectra of the imipenem hydrolysis assay with a carbapenem-sensitive *A. baumannii* strain. Incubation at 37°C during 4 h; NaCl 0.45%; imipenem concentration 0.25 mg/mL Units of the *x* axis represent the mass per charge in Daltons [m/z (Da)] and that of the *y* axes, the relative intensity (a.u., arbitrary units).

Kinetic studies of imipenem degradation were performed for the three carbapenem-resistant *K. pneumoniae* reference strains (one bla_KPC_ and two bla_NDM-1_). These strains were incubated in the presence of imipenem at a concentration of 0.25 mg/mL for up to four hours, and the results indicate that the three strains completely degraded imipenem within 15 min. The peak at 300.0 m/z completely vanished whereas the peak at 254.0 m/z increased during the experiment. Experiments performed with the *E. coli* ATCC 25922 reference strain confirmed that this strain does not contain carbapenemase activity, as both the peak at 300.0 m/z and at 254.0 m/z were still present after 4 hours of incubation. Kinetic studies were also performed on eight *A. baumannii* strains (six strains resistant to imipenem and two strains sensitive to imipenem i.e strain SDF and strain AYE) at an imipenem concentration of 0.25 mg/mL and an incubation time of 2 hours. With these parameters, the peak at 300.0 m/z disappeared for each of the carbapenem-resistant strains (Figure 2) except one with an increase of the area under curve for the peak at 254.0 m/z. Interestingly, although the carbapenem-resistant strain for which the peak at 300.0 m/z did not completely disappear after 2 hours, the ratio of the area of the two peaks at 2 hours was <0.5, and the peak disappeared using 4 hours of incubation. Finally, for the two carbapenem-sensitive strains, the peak at 300.0 m/z was consistently present during the test both after 2 hours and 4 hours of incubation (Figure 3), and the ratio of the area of imipenem/metabolite was >0.5, confirming that these strains do not contain carbapenemase activity.

The imipenem hydrolysis assay was performed blindly twice on the 149 strains listed in Table 1 using the two criteria listed above, i.e., the disappearance of the peak at 300.0 m/z along with an increase of the peak at 254.0 m/z leading to a ratio of the area between the two peaks <0.5, to identify carbapenemase activity. These strains included 63 carbapenem-resistant *A. baumannii* strains containing either the bla_OXA23-like_ and/or bla_OXA24-like_ carbapenemase-encoding genes (46 from Algeria and 17 from Marseille, France), 43 carbapenem-sensitive *A. baumannii* strains (42 from Algeria and one from Marseille, France), 35 carbapenem-sensitive *K. pneumoniae* strains (31 ESBL producers with CTX-M15-, TEM- and SHV-encoding genes and four non-ESBL producers), and the eight control strains listed above (Table 1). Results showed that after 2 hours of incubation time, a carbapenemase activity was observed for all the carbapenem-resistant control strains tested, with disappearance of the peak at 300 m/z and a ratio of the area of imipenem/metabolite <0.5. Concerning the 63 carbapenem-resistant *A. baumannii* strains disappearance of the peak at 300 m/z was observed after 2 hours of incubation for 60 strains and was achieved for all strains after 4 hours of incubation (see additional examples in Figure S2). Concerning the carbapenem-sensitive controls, no carbapenemase activity was detected after 2 or 4 hours of incubation (presence of the peak at 300 m/z and ratio of the area of imipenem/metabolite >0.5). Similarly, the 43 carbapenem-sensitive *A. baumannii* strains showed the presence of the peak at 300 m/z (see additional examples in Figure S2) and a ratio of the area of imipenem/metabolite >0.5 whatever the time of incubation tested. Thus in all experiments, disappearance of the peak at 300 m/z was correlated with a ratio <0.5 (Table S1). The overall sensitivity and specificity for the detection of carbapenemase activity in these 106 *A. baumannii* strains were 95.2% and 100.0%, respectively after two hours of incubation with imipenem, and 100.0% and 100.0% respectively, after four hours of incubation. Thus, a concentration of 0.25 mg/L of imipenem and an incubation time of 4 hours were the optimal conditions.

## Discussion

The MALDI-TOF MS technique has recently been introduced as a fast and reliable identification method that can be used for routine applications in diagnostic laboratories [18]. This rapid, simple, inexpensive and high-throughput proteomic system has been shown to be useful in bacteria and yeast identification [19]. This method has also commonly been used for proteomic research at the molecular level [20]. In our study, the MALDI-TOF MS system was used for the detection of carbapenemase activity in a large collection of *A. baumannii* strains isolated in France and Algeria. The strains were characterized according to their resistance mechanisms using phenotypic tests, including antibiotic susceptibility testing on agar plates, and also using genotypic tests, including the search for oxacillinases- and Ambler class B β-lactamase-encoding genes.

The direct detection of carbapenemase activity using similar approaches involving MALDI-TOF was recently reported in two studies of *P. aeruginosa* and *Enterobacteriaceae* [16], [17], but to our knowledge, this was the first time that the direct detection of carbapenemase activity was developed using imipenem as the carbapenem compound and including a large collection of *A. baumannii* clinical isolates and other bacterial strains (a total of 149 bacterial strains). Indeed, in these two previous studies, either ertapenem [16] or meropenem [16] was used to detect carbapenemase activity for 87 bacterial isolates (including 47 carbapenem-resistant strains) and 124 bacterial isolates (including 30 carbapenem-resistant strains), respectively. However, the MALDI-TOF assay used in the current study was different from that used in these two previous reports. First, in this study, we used an Ultraflex I mass spectrometer, which has greater resolution and greater sensitivity than the Microflex mass spectrometer that was used previously [16], [17]. The Ultraflex is a more reliable instrument because it contains 2 tubes for flight with a total of 2.76 meters versus only one tube of 0.95 meters for the Microflex apparatus. Moreover, the Ultraflex provides improved resolution of peaks when the positive reflectron ion mode is used between m/z 0 and 1000 Da (Data Bruker Daltonics). Finally, the Ultraflex has increased sensitivity when AnchorChip target plates covered with a hydrophobic surface with a hydrophilic center are used, which also leads to a higher concentration of the molecules to be ionized. Second, before testing clinical isolates, we evaluated different matrices to optimize the detection of imipenem and to limit the noise due to the matrix itself. Our results indicated that the HCCA matrix diluted in a mixture of acetone, ethanol and TFA was ideal to visualize the imipenem mass spectra with the Ultraflex instrument, as the background noise was low for this condition. The peak of imipenem molecule can be visualized at 300.0+/−0.2 m/z, as well as the natural degradation product at 254.0+/−0.1 m/z. The presence of the peak of the metabolite at 254 m/z even in the absence of any bacterial colonies could be explained by the fact that there is a spontaneous degradation of imipenem in sodium chloride [21]. This was also observed by Burckhardt *et al* with ertapenem (476 Da) and its natural hydrolyzed and decarboxylated ertapenem metabolite (450 Da) [16]. In order to consider this low amount of metabolite we have standardized our assay with calculation of the ratio of area under curves between imipenem and its metabolite to take this phenomenon into account. These peaks were highly reproducible and corresponded to the masses of the analytes studied, which allowed for the confirmation of their identities. Third, the method used in this study (Ultraflex machine+AnchorChip target+HCCA matrix) had the benefit that only the native imipenem molecule and its metabolite were detected without the sodium salt variants that were found with the Microflex method [16], [17]. Thus, using our method, a carbapenem-sensitive strain could easily be identified by the presence of the peak at 300.0 m/z after 4 hours, and a carbapenem-resistant strain could be identified by the disappearance of this peak after 4 hours of incubation. Interestingly, we observed the same phenomenon with meropenem. However, imipenem was preferable over meropenem for this assay because it has been shown to be more sensitive and specific for the phenotypic detection of carbapenem-resistant strains. In addition, in our hands, we observed an overlapping peak between the meropenem antibiotic at 384.1 m/z and the matrix at 380.0 m/z (data not shown).

The use of this MALDI-TOF carbapenemase detection assay for the evaluation of *A. baumannii* clinical isolates was easy and efficient and demonstrated both high sensitivity and specificity. Similar sensitivity and specificity results were reported regarding the use of meropenem for carbapenemase detection in *Enterobacteriaceae* and *P. aeruginosa.* [17]. Among the 43 carbapenem-susceptible and 63 carbapenem-resistant *A. baumannii* strains tested, neither false-positive nor false-negative result was observed. Interestingly, one of the three carbapenem-resistant clinical isolates of *A. baumannii* for which the peak at 300.0 m/z did not completely disappear after 2 hours but only after 4 hours of incubation was an isolate that had acquired resistance to colistin and that is believed to have a reduced fitness [22], likely explaining the delayed disappearance of this peak, which persisted up to 2 hours. Regarding the control strains, neither false-positive nor false-negative results were noted. For all of the tested carbapenem-sensitive strains, the imipenem peak was clearly distinguishable at 300.0 m/z regardless of the incubation time tested and the ratio of the area of imipenem/metabolite was strictly >0.5. For the carbapenem-resistant strains, the peak at 300.0 m/z disappeared for all strains in all cases and the ratio of the area of imipenem/metabolite was strictly <0.5.

Finally, this study found that the delayed degradation of imipenem varied according to carbapenemase type and not to the MIC or to the ability of the bla_NDM-1_ and bla_KPC_ carbapenemases from *K. pneumoniae* to hydrolyze imipenem faster (<30 min) than the bla_OXA-23-like_- or bla_OXA-24-like_ carbapenemases from *A. baumannii* (≤2.5 h). Our results corroborate those of Burckhardt *et al.,* who found that ertapenem was hydrolyzed in 1 h by the bla_NDM-1_ and bla_KPC_ carbapenemases and in 1.5 to 2.5 h by the bla_IMP_ and bla_VIM_ carbapenemases [16]. This difference in the time required for complete imipenem hydrolysis suggests that either the oxacillinases from *A. baumannii* have weaker carbapenemase activity/affinity for imipenem or that *A. baumannii* grows more slowly than *Enterobacteriaceae* species. The detection of carbapenem resistance using the MALDI-TOF MS method has many advantages over other techniques, such as PCR, because it can detect low-level carbapenemase activity at a low cost even when the causative enzyme is unknown. Therefore, this technique is suitable both for the rapid detection of resistance in clinical settings and for the discovery of new carbapenemases. However, one of the disadvantages of this method is that it can detect only enzymatic carbapenem resistance and not resistance due to efflux mechanisms or porin alterations in *A. baumannii* [16]. However, because this assay was able to detect specific peaks corresponding to the exact mass of the specific drug, we believe that all enzymatic antibiotic resistance mechanisms could be rapidly detected using the method presented herein.

In conclusion, because the average turnaround time for this test was estimated to be 4 h, our study clearly demonstrates that this assay could be used in real-time for routine use in clinical microbiology laboratories as a point-of-care strategy to identify carbapenemase-producing *A. baumannii* strains, which would aid in the prevention of outbreaks and the spread of uncontrollable superbugs.

## Materials and Methods

### Bacterial strains and carbapenemase detection

The carbapenem-resistant and carbapenem-sensitive *A. baumannii* strains used in this study originated from the collection of the Department of Microbiology at the University Hospital of Marseille (France) and from Tlemcen, Setif, Sidi Bel Abbes, Oran and Tizi Ouzou (Algeria) (Table 1). Species were identified using the Bruker Daltonics Ultraflex MALDI-TOF MS method (Bremen, Germany), as previously described [23]. Susceptibility results for each *A. baumannii* strain were determined using the disc diffusion method. The MIC for imipenem was determined using the E-test method and was interpreted according to the guidelines recommended by the Comité de l'Antibiogramme de la Société Française de Microbiologie (CA-SFM) (www.sfm-microbiologie.org/). For each of the *A. baumannii* strains, genes encoding the Ambler class B and D carbapenemases were identified by PCR with primers specific for the *bla*_IMP_, *bla*_VIM_, *bla*_NDM_, *bla*_OXA-23-like_, *bla*_OXA-24-like_, *bla*_OXA-51-like_ and *bla*_OXA-58-like_ genes as previously described [24]. Four carbapenem-resistant *P. aeruginosa* strains (two bla_VIM_ and two bla_IMP_), three carbapenem-resistant *Klebsiella pneumoniae* strains (one bla_KPC_ and two bla_NDM-1_), one carbapenem-susceptible *E. coli* reference strain ATCC 25922, and 35 carbapenem-sensitive *K. pneumoniae* strains (31 ESBL producers and four non-ESBL producers) were used as controls (Table 1). For kinetic studies, the carbapenem-sensitive *A. baumannii* reference strains AYE and SDF [25] were used as well as six well characterized carbapenem-resistant strains.

### MALDI-TOF MS analysis of imipenem

Commercially available imipenem that contains cilastatin (Tienam, 500 mg, MSD, Paris, France) was diluted in 0.45% NaCl. The MALDI-TOF analysis was performed using the spectra of low molecular masses ranging from 0 to 1000 Da. Due to the background peaks from the low molecular masses of the matrix, three organic matrices were tested: 1) 10 mg/ml α-cyano-4-hydroxycinnamic acid (HCCA); 2) 2,5-dihydroxybenzoic acid (DHB) diluted in acetonitrile and water (1/1) and 3) 3.3 mg/ml of α-cyano-4-hydroxycinnamic acid (HCCA) diluted in a mixture of acetone, ethanol and TFA (1/2) (all reagents were obtained from Sigma-Aldrich, Lyon, France).

One microliter of the matrix solution was mixed with one microliter of the sample, which was applied onto a target (Bruker Daltonics GmbH, Bremen, Germany; MTP AnchorChip™ 384 T F Target) and allowed to dry at room temperature. Mass spectra were acquired using an Ultraflex I mass spectrometer and the flexControl 3.0 software (Bruker Daltonics GmbH) operating in positive reflection ion mode between m/z 0 and 1000 Da. The parameters were set as follows: ion source 1: 25 kV; ion source 2: 21.5 kV; lens: 10 kV; reflector 1: 25.5 kV; reflector 2: 14.19 kV; pulsed ion extraction: 10 ns; and detection gain: 9.1×. A total of 500 shots were acquired in 5 different positions for one spectrum.

### Standardization and internal calibration of the Ultraflex I mass spectrometer

The imipenem concentrations that were tested for calibration of the mass spectrometer ranged from 0.25 mg/mL to 2 mg/mL. For internal calibration of the Ultraflex mass spectrometer, we established the mass spectrum of pure imipenem that also contains a natural degradation product during time as demonstrated by Swanson *et al* [21]. The theoretical peaks of imipenem and its natural degradation product were calculated using ISIS Draw software and were used for internal calibration of the Ultraflex apparatus in a set of 200 independent experiments. Once these peaks were determined, they were used as internal calibration controls for the imipenem hydrolysis assay. Finally, stability of imipenem molecule was checked in a 6 hours incubation assay.

### Imipenem hydrolysis assay

Cultures of the *A. baumannii* strains and the controls were incubated overnight on blood agar plates (bioMérieux, Lyon, France) at 37°C. Then, a 10-µl loop-sized amount of bacteria was added to 1 mL 0.45% NaCl, as previously described by Burckhardt *et al.* [16], with or without imipenem at concentrations ranging from 0.25 mg/mL to 2 mg/mL, and the cultures were incubated for up to 4 h at 37°C. The tubes were then centrifuged for 3 min at 12,000× g, and 1 µl of the clear supernatant was applied to each target spot, mixed with one microliter of matrix solution and left to dry at room temperature. After preparing and validating the analytical and technical aspects of the assay (incubation time, concentration to be used, reproducibility of the drug and metabolite peaks), all tests with clinical isolates were conducted blindly and in duplicate. Two spots of each clinical isolate were performed in all experiments.

### Spectra analysis and interpretation of carbapenemase activity

For one spectrum, approximately 500 shots were totaled. The result was interpreted as positive for carbapenemase production if the specific peak for imipenem (300.0 m/z, see results section) disappeared completely during the incubation time and if the peak of the natural metabolite at 254.0 m/z (see results section) increased as measured by the area under the curves leading to a ratio between the peak for imipenem and its metabolite becoming <0.5.

## Supporting Information

Figure S1 **Mass spectra of pure imipenem showing the stability of the drug (presence of the specific peak at 300 m/z) during a 6 hours incubation time.**
(EPS)

Figure S2 **Mass spectra of 24 clinical isolates of** ***A. baumannii*** obtained after 4 hours of incubation showing the disappearance of the peak at 300 m/z for resistant isolates (n = 15) and the persistence of this peak for susceptible isolates (n = 9). Strain numbers (S.) are those presented as * in Table S1.
(PDF)

Table S1 Area under curves (AUC) and ratio between imipenem peak and its metabolite for the 106 *Acinetobacter baumannii* clinical strains according to their location and phenotype of resistance to imipenem. R = resistant; S = susceptible. * = strains for which mass spectra at 300 m/z are provided in Figure S1.
(DOC)
