# Supplementary figures and images for: Rapid Detection of Carbapenem Resistance in Acinetobacter baumannii Using Matrix-Assisted Laser Desorption Ionization-Time of Flight Mass Spectrometry
Source: PLoS One. 2012 Feb 16;7(2):e31676. doi: 10.1371/journal.pone.0031676 (PMC3280980; doi:10.1371/journal.pone.0031676)

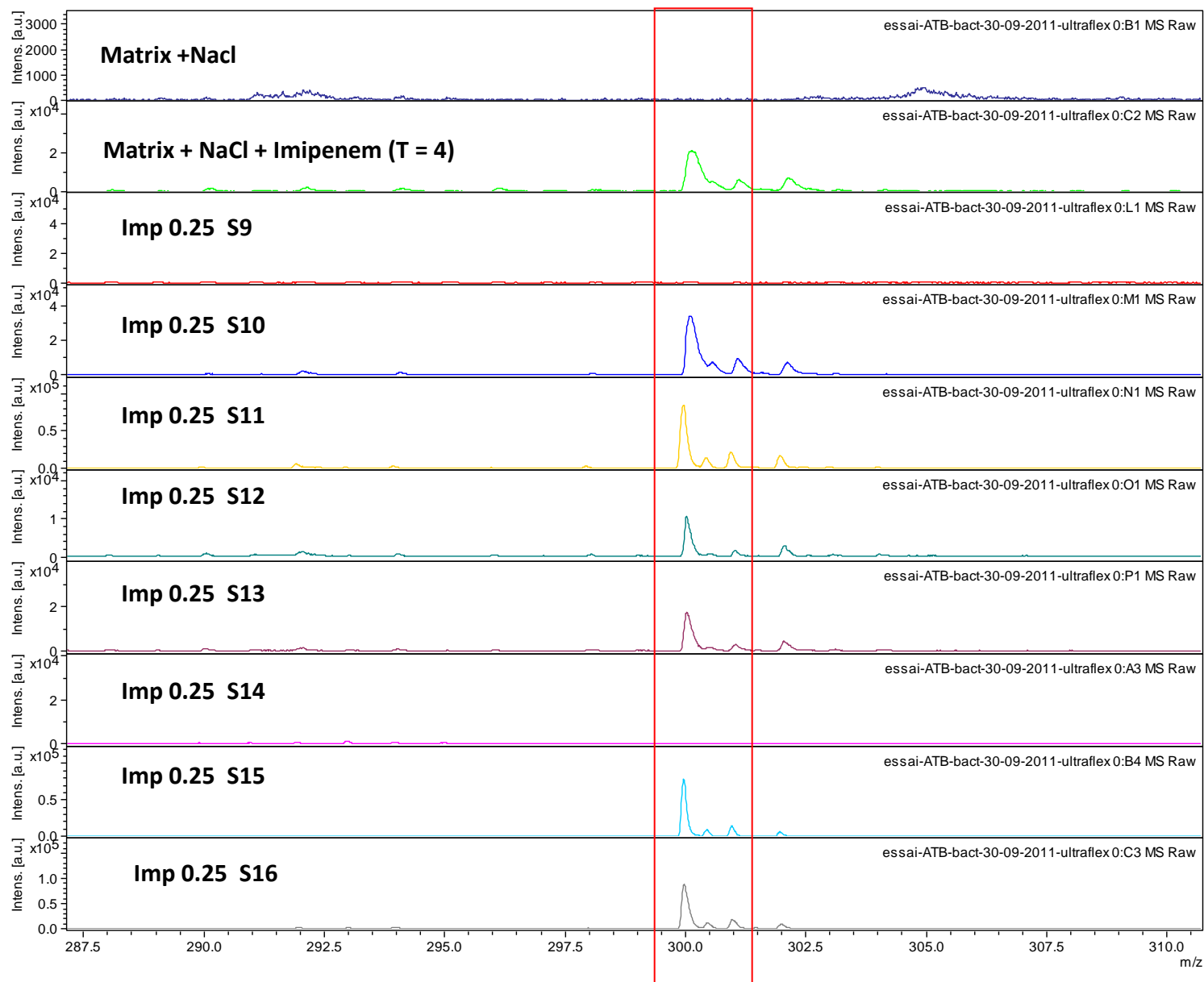

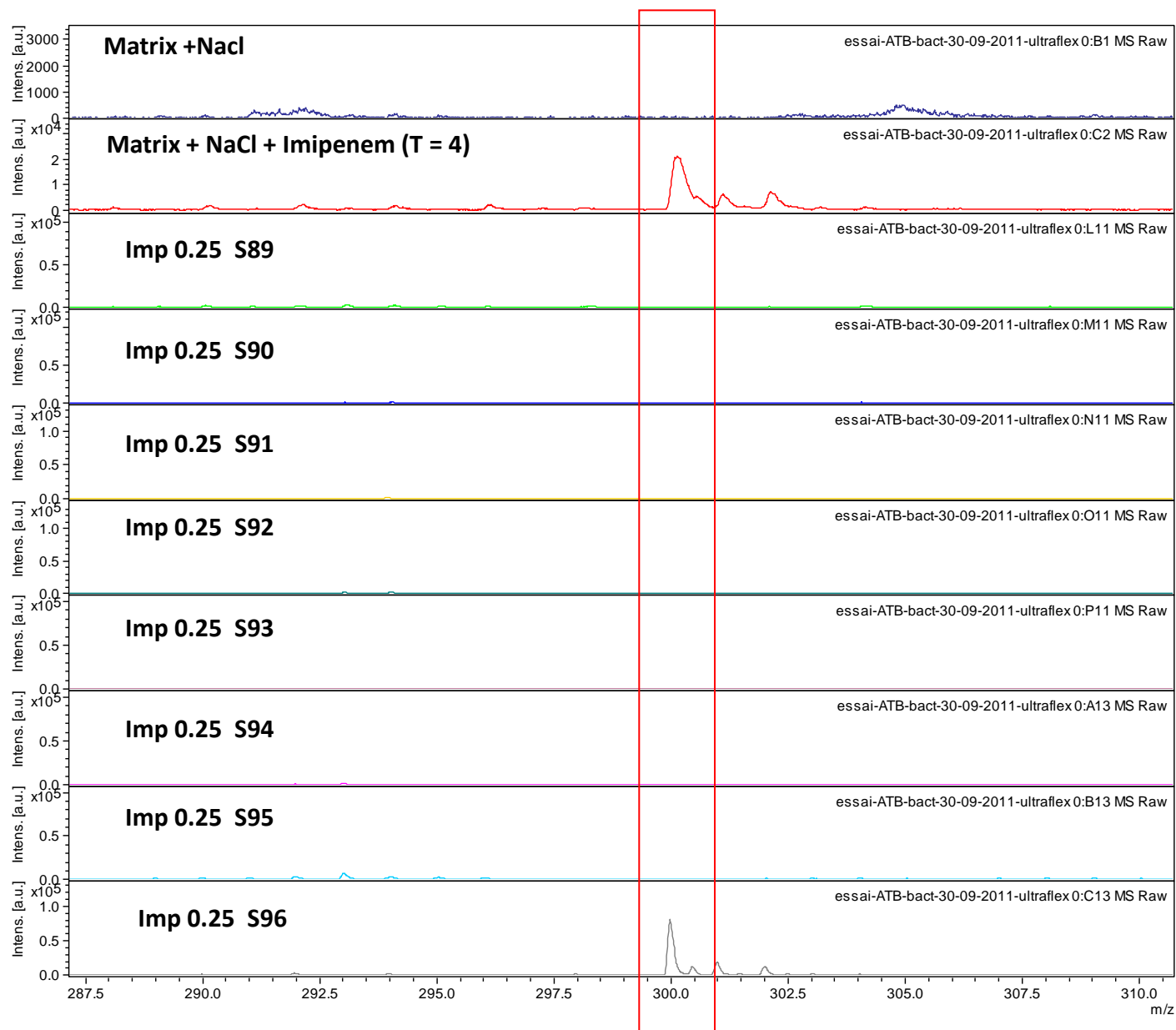

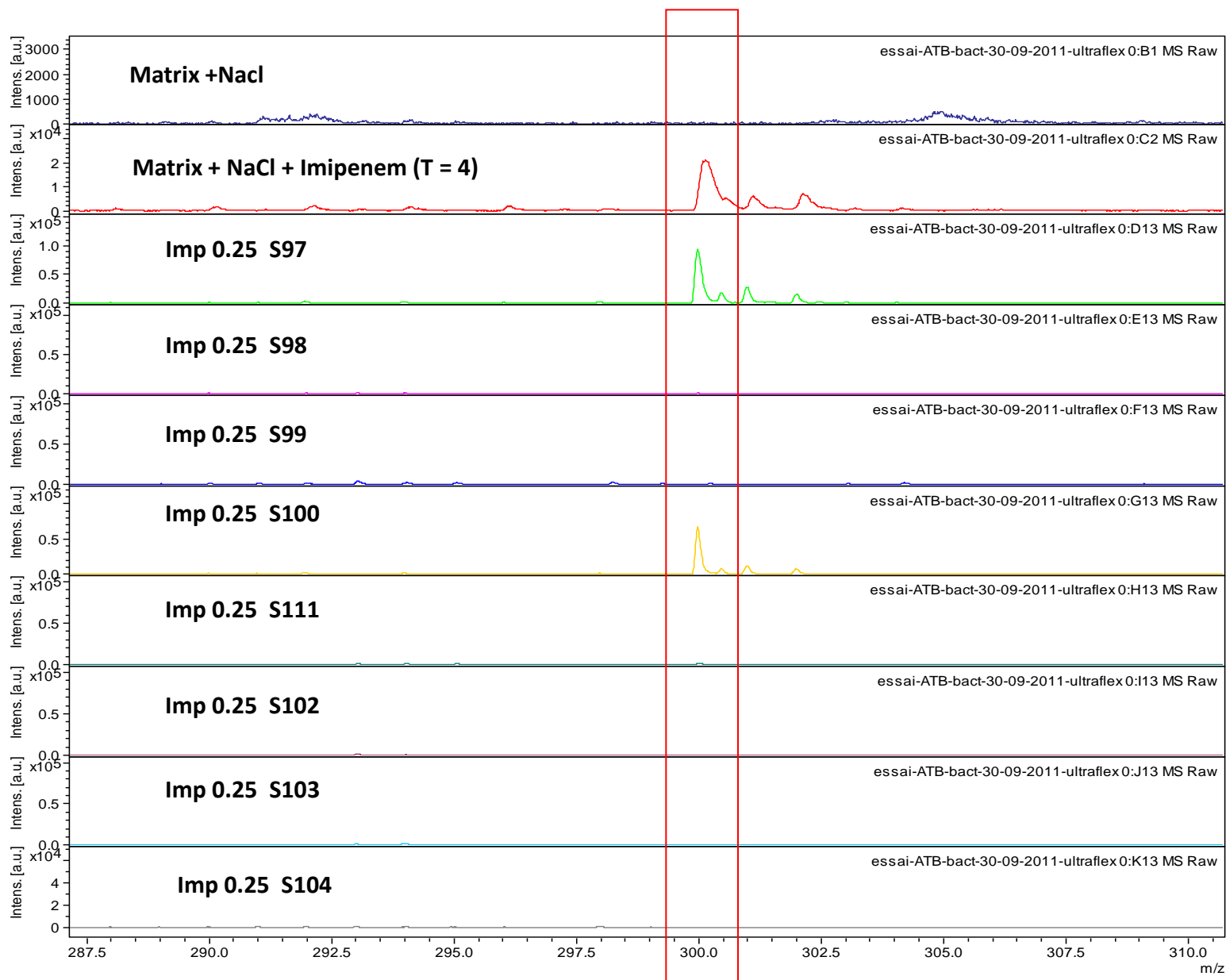

Supplement: Figure S2 — Mass spectra of 24 clinical isolates of A. baumannii obtained after 4 hours of incubation showing the disappearance of the peak at 300 m/z for resistant isolates (n = 15) and the persistence of this peak for susceptible isolates (n = 9). Strain numbers (S.) are those presented as * in Table S1. (PDF) [file pone.0031676.s002.pdf]
