# Supplementary material for: Rapid Detection of Carbapenem Resistance in Acinetobacter baumannii Using Matrix-Assisted Laser Desorption Ionization-Time of Flight Mass Spectrometry
Source: PLoS One. 2012 Feb 16;7(2):e31676. doi: 10.1371/journal.pone.0031676 (PMC3280980; doi:10.1371/journal.pone.0031676)
Supplement: Table S1 — Area under curves (AUC) and ratio between imipenem peak and its metabolite for the 106 Acinetobacter baumannii clinical strains according to their location and phenotype of resistance to imipenem. R = resistant; S = susceptible. * = strains for which mass spectra at 300 m/z are provided in Figure S1. (DOC) [file pone.0031676.s003.doc]

**Supplementary Table 1**. Area under curves (AUC) and ratio between imipenem peak and its metabolite for the 106 *Acinetobacter baumannii* clinical strains according to their location and phenotype of resistance to imipenem. R = resistant; S = susceptible. * = strains for which mass spectra at 300 m/z are provided in Supplementary Figure 1.

| **City** | **Phenotype** | **Strain Number** |  | **AUC IMP** | **AUC metabolite** | **ratio** |
| --- | --- | --- | --- | --- | --- | --- |
| Sétif | **R** | **1** |  | 0 | 3616 | 0 |
| Sétif | **R** | **2** |  | 0 | 4453 | 0 |
| Sétif | **R** | **4** |  | 0 | 2980 | 0 |
| Tizi Ouzou | **R** | **14*** |  | 0 | 4070 | 0 |
| Sétif | **R** | **17** |  | 0 | 4953 | 0 |
| Sétif | **R** | **20** |  | 0 | 4910 | 0 |
| Marseille | **R** | **21** |  | 0 | 1862 | 0 |
| Sétif | **R** | **22** |  | 0 | 3803 | 0 |
| Sétif | **R** | **23** |  | 0 | 7991 | 0 |
| Marseille | **R** | **24** |  | 0 | 7408 | 0 |
| Sétif | **R** | **27** |  | 0 | 2654 | 0 |
| Sidi Bel Abbes | **R** | **29** |  | 0 | 810 | 0 |
| Sétif | **R** | **30** |  | 0 | 9734 | 0 |
| Tlemcen | **R** | **31** |  | 0 | 5751 | 0 |
| Sétif | **R** | **35** |  | 0 | 13269 | 0 |
| Marseille | **R** | **36** |  | 0 | 5881 | 0 |
| Marseille | **R** | **37** |  | 0 | 8745 | 0 |
| Marseille | **R** | **38** |  | 0 | 6797 | 0 |
| Sétif | **R** | **39** |  | 0 | 6922 | 0 |
| Tizi Ouzou | **R** | **42** |  | 0 | 3985 | 0 |
| Sidi Bel Abbes | **R** | **45** |  | 0 | 5640 | 0 |
| Sétif | **R** | **48** |  | 0 | 3863 | 0 |
| Sétif | **R** | **49** |  | 0 | 6322 | 0 |
| Sétif | **R** | **50** |  | 0 | 10476 | 0 |
| Tlemcen | **R** | **52** |  | 0 | 3488 | 0 |
| Tizi Ouzou | **R** | **53** |  | 0 | 12576 | 0 |
| Marseille | **R** | **57** |  | 0 | 13967 | 0 |
| Sétif | **R** | **58** |  | 0 | 8489 | 0 |
| Sétif | **R** | **63** |  | 0 | 10197 | 0 |
| Oran | **R** | **64** |  | 0 | 3692 | 0 |
| Marseille | **R** | **67** |  | 0 | 13556 | 0 |
| Sétif | **R** | **68** |  | 0 | 9081 | 0 |
| Marseille | **R** | **69** |  | 0 | 8557 | 0 |
| Sétif | **R** | **70** |  | 0 | 12426 | 0 |
| Sétif | **R** | **75** |  | 0 | 10215 | 0 |
| Tlemcen | **R** | **76** |  | 0 | 5462 | 0 |
| Sétif | **R** | **81** |  | 0 | 3957 | 0 |
| Sétif | **R** | **83** |  | 0 | 5454 | 0 |
| Sétif | **R** | **87** |  | 0 | 13129 | 0 |
| Marseille | **R** | **88** |  | 0 | 8404 | 0 |
| Marseille | **R** | **89*** |  | 0 | 13308 | 0 |
| Sétif | **R** | **90*** |  | 0 | 11584 | 0 |
| Sétif | **R** | **91*** |  | 0 | 12240 | 0 |
| Sétif | **R** | **92*** |  | 0 | 9964 | 0 |
| Sétif | **R** | **93*** |  | 0 | 4880 | 0 |
| Sétif | **R** | **94*** |  | 0 | 6589 | 0 |
| Marseille | **R** | **95*** |  | 0 | 11219 | 0 |
| Tlemcen | **R** | **99*** |  | 0 | 7701 | 0 |
| Marseille | **R** | **102*** |  | 0 | 6564 | 0 |
| Sétif | **R** | **103*** |  | 0 | 10232 | 0 |
| Sétif | **R** | **104*** |  | 0 | 6135 | 0 |
| Oran | **R** | **108** |  | 0 | 3233 | 0 |
| Tlemcen | **R** | **109** |  | 0 | 4438 | 0 |
| Oran | **R** | **112** |  | 0 | 4781 | 0 |
| Marseille | **R** | **34** |  | 29 | 4084 | 0,01 |
| Oran | **R** | **98*** |  | 173 | 8507 | 0,02 |
| Marseille | **R** | **8** |  | 184 | 5728 | 0,03 |
| Sétif | **R** | **33** |  | 57 | 2214 | 0,03 |
| Marseille | **R** | **55** |  | 155 | 6078 | 0,03 |
| Tlemcen | **R** | **111*** |  | 272 | 7844 | 0,03 |
| Marseille | **R** | **9*** |  | 87 | 1848 | 0,05 |
| Sétif | **R** | **66** |  | 433 | 6908 | 0,06 |
| Marseille | **R** | **7** |  | 925 | 4100 | 0,23 |

| **City** | **Phenotype** | **Strain Number** |  | **AUC IMP** | **AUC metabolite** | **ratio** |
| --- | --- | --- | --- | --- | --- | --- |
| Tizi Ouzou | **S** | **54** |  | 2711 | 2798 | 0,97 |
| Tizi Ouzou | **S** | **47** |  | 7642 | 7708 | 0,99 |
| Oran | **S** | **61** |  | 7268 | 7327 | 0,99 |
| Tlemcen | **S** | **12*** |  | 3735 | 3761 | 0,99 |
| Tizi Ouzou | **S** | **3** |  | 5039 | 5039 | 1 |
| Tizi Ouzou | **S** | **73** |  | 3766 | 3600 | 1,05 |
| Tizi Ouzou | **S** | **82** |  | 6538 | 5685 | 1,15 |
| Tizi Ouzou | **S** | **6** |  | 5978 | 5193 | 1,15 |
| Tizi Ouzou | **S** | **72** |  | 4855 | 4204 | 1,15 |
| Sidi Bel Abbes | **S** | **11*** |  | 1798 | 1540 | 1,17 |
| Tizi Ouzou | **S** | **5** |  | 7875 | 6605 | 1,19 |
| Sétif | **S** | **10*** |  | 4984 | 4081 | 1,22 |
| Tizi Ouzou | **S** | **44** |  | 3120 | 2536 | 1,23 |
| Tizi Ouzou | **S** | **26** |  | 5139 | 4124 | 1,25 |
| Tizi Ouzou | **S** | **107** |  | 1998 | 1554 | 1,29 |
| Tizi Ouzou | **S** | **71** |  | 5538 | 4220 | 1,31 |
| Sétif | **S** | **65** |  | 8450 | 5784 | 1,46 |
| Tlemcen | **S** | **101** |  | 21009 | 14212 | 1,48 |
| Tlemcen | **S** | **74** |  | 7905 | 5285 | 1,5 |
| Tlemcen | **S** | **41** |  | 3642 | 2229 | 1,63 |
| Marseille | **S** | **18** |  | 13822 | 8034 | 1,72 |
| Tizi Ouzou | **S** | **105** |  | 5997 | 3476 | 1,73 |
| Tizi Ouzou | **S** | **59** |  | 7453 | 3987 | 1,87 |
| Tizi Ouzou | **S** | **60** |  | 13973 | 7434 | 1,88 |
| Sétif | **S** | **77** |  | 4335 | 2285 | 1,9 |
| Sétif | **S** | **85** |  | 11018 | 5605 | 1,97 |
| Tizi Ouzou | **S** | **96*** |  | 12632 | 6356 | 1,99 |
| Tizi Ouzou | **S** | **25** |  | 16213 | 7229 | 2,24 |
| Sétif | **S** | **13*** |  | 12490 | 5506 | 2,27 |
| Sétif | **S** | **19** |  | 12490 | 5506 | 2,27 |
| Sétif | **S** | **86** |  | 10077 | 4325 | 2,33 |
| Tizi Ouzou | **S** | **100*** |  | 9129 | 3909 | 2,34 |
| Tizi Ouzou | **S** | **79** |  | 6022 | 2520 | 2,39 |
| Sétif | **S** | **80** |  | 12390 | 4905 | 2,53 |
| Sétif | **S** | **32** |  | 3843 | 1476 | 2,6 |
| Tizi Ouzou | **S** | **15*** |  | 15874 | 5781 | 2,75 |
| Tizi Ouzou | **S** | **46** |  | 5581 | 1979 | 2,82 |
| Tizi Ouzou | **S** | **16*** |  | 7639 | 2672 | 2,86 |
| Oran | **S** | **43** |  | 18167 | 6341 | 2,87 |
| Tizi Ouzou | **S** | **40** |  | 15710 | 5294 | 2,97 |
| Tizi Ouzou | **S** | **97*** |  | 16650 | 5449 | 3,06 |
| Tizi Ouzou | **S** | **84** |  | 11480 | 2557 | 4,49 |
| Sétif | **S** | **106** |  | 12655 | 2553 | 4,96 |
